# Sea ice dynamics structure narwhal presence and seasonal movements in a Northwest Greenland fjord system

**DOI:** 10.1038/s41598-026-53787-8

**Published:** 2026-05-21

**Authors:** Julie Sofie K. Larsen, Michael Ladegaard, Peter T. Madsen, Steffen M. Olsen, Malene Simon

**Affiliations:** 1 Greenland Climate Research Centre, Greenland Institute of Natural Resources, Nuuk, Greenland; 2 Zoophysiology, Department of Biology, Aarhus University, Aarhus, Denmark; 3 Danish Meteorological Institute (DMI), Copenhagen, Denmark

**Keywords:** *Monodon monoceros*, Sea ice, Marginal ice zone, Passive acoustic monitoring, Arctic wildlife, North water polynya, Qaanaaq, Climate sciences, Ecology, Ecology, Ocean sciences

## Abstract

The Arctic is experiencing unprecedented environmental change, with diminishing sea ice reshaping marine ecosystems. The narwhal (*Monodon monoceros*) exhibits strong site fidelity during seasonal movements and continued sea-ice decline has the potential to alter important habitats. This study aimed to understand narwhal presence at different sea-ice stages by combining long-term passive acoustic monitoring (PAM) with satellite-derived sea ice data from Inglefield Bredning, Northwest Greenland (June 2022–September 2025). Generalized linear mixed models (GLMMs) revealed a highly significant effect of sea-ice stage on narwhal acoustic activity (*p* < 0.001). Activity peaked during partial ice cover (β = 1.73 ± 0.17, *p* < 0.001) but declined sharply under open-water and freeze-up conditions (β = − 0.98 ± 0.19, *p* < 0.001). These results demonstrate that narwhal presence is closely synchronized with sea-ice timing and extent, emphasizing the marginal ice zone as a key ecological feature that narwhals exploit, likely because it provides both feeding opportunities and refuge from predators. Continued loss of seasonal sea ice is therefore expected to alter narwhal movement patterns, potentially increasing fjord residency and reducing inter-fjord movements. Such behavioural shifts could have cascading ecological effects, altering prey dynamics and reducing genetic exchange among regional narwhal groups.

**Supplementary Information:**

The online version contains supplementary material available at 10.1038/s41598-026-53787-8.

## Introduction

Sea ice is a dynamic and defining element of Arctic marine ecosystems^1^. Its seasonal formation and retreat have been shown to structure predator-prey interactions, influence access to foraging habitats, and mediate exposure to both natural and anthropogenic disturbances^2,3^. Yet despite this central role, there remains limited knowledge of how these changing ice conditions affect the timing of seasonal movements in species relying on sea ice for key aspects of foraging, reproduction, or safe movement^4^. Changes in Arctic sea ice have the potential to alter habitat use, distribution, and foraging^2,5^, making it particularly important to understand how species respond to these shifting physical constraints as Arctic sea ice becomes increasingly unpredictable in space and time^1,6^.

The narwhal (*Monodon monoceros*) is an Arctic species that inhabits sea ice environments primarily across Greenland and Canada, exhibiting strong site fidelity and predictable seasonal movements between coastal summering grounds and offshore winter habitats across their range^3,7,8^. Narwhals are commonly described as occurring in distinct subpopulations defined by the fjords and bays where they aggregate during summer^9^. In several Greenlandic fjords, marine terminating glacier fronts drive upwelling of nutrient-rich deeper waters to the euphotic zone and create productive feeding areas that attract narwhals during the open-water season, generally from May to November^7,10,11^. Narwhals may also select these environments for their thermal and bathymetric conditions, potentially reflecting prey distributions^11,12^. Inglefield Bredning is a glacial fjord in Northwest Greenland used as a narwhal summering habitat, supporting a subpopulation estimated at approximately 8300 individuals^13^. This subpopulation is believed to migrate to the North Water Polynya (Pikialasorsuaq), a recurring area of open water that persists throughout winter^13,14^. However, it remains uncertain whether this group forms a distinct population or mixes with narwhals from Canadian summering grounds. Several subpopulations share offshore wintering areas^8,15^. Although mating is thought to occur during the spring migration toward summering grounds^16^, the timing and location of reproduction remain uncertain, and movements between wintering and summering areas are not fully understood (i.e., due to limited movement data and logistical constraints), which limits our ability to predict how narwhals use their summer habitats and respond to changing sea-ice conditions. Information is also lacking on whether narwhals have a continuous presence near the fjord entrance year-round, or whether they leave this area entirely during parts of the Arctic winter or when the inner fjord becomes accessible.

While glacial fronts provide well-documented foraging opportunities in the summer^8,10,11^, sea-ice conditions may impact narwhal movements and behaviours within fjords^17^. In particular, sea ice can restrict or open access to certain habitats, modulate exposure to predators such as killer whales (*Orcinus orca*), and provide productive habitat for forage species such as polar cod (*Boreogadus saida*) that are known to be attracted to ice edges^2,17–21^. Sea ice is expected to create barriers that influence when and how narwhals can access remote fjord areas, with some ice edges potentially serving more as navigational routes or stop-over sites^22^, reflecting variation in ice-associated habitat use. However, whether sea-ice conditions directly influence the timing of arrival and departure of narwhals has not previously been investigated.

If sea ice indeed plays an active role in shaping when and how narwhals use fjords, ongoing reductions in ice extent, thickness, and seasonal duration due to rapid Arctic warming^1,23–25^, may alter their seasonal presence and habitat use. Changes in sea-ice phenology could alter the timing and duration of narwhal presence within fjords^12,26,27^, increase exposure to human disturbance such as vessel traffic, and modify interactions with prey and predators^24,28,29^. These environmental changes also have implications for Indigenous communities who depend on Arctic marine mammals for subsistence, emphasizing the intertwined ecological and socio-cultural significance of sea ice^25,30^. Understanding how the timing and extent of sea-ice formation and break-up influence narwhal movements is therefore essential to clarify the drivers of seasonal presence and absence and to guide conservation and ecosystem management.

In this study, we investigate how changes in sea-ice cover, both in concentration and timing, affect narwhal presence in the outer parts of the Inglefield Bredning fjord system. Collecting passive acoustic monitoring (PAM) data across time periods with changing and unstable sea-ice conditions can be logistically challenging. By deploying acoustic loggers biannually through landlocked sea ice in spring, we overcame typical data logger longevity constraints and continuously recorded narwhal presence from arrival to departure. We combine long-term PAM data from Inglefield Bredning with satellite-derived sea ice data to quantify how sea-ice conditions affect narwhal presence and habitat-use within the fjord. Narwhals are well known for their ability to navigate dense sea-ice conditions, yet they also face the risk of becoming trapped if a fjord freezes rapidly. This raises the question of how closely their movements into fjords are linked to changes in sea ice. In this study, we investigate whether narwhals are present year-round near the entrance to Inglefield Bredning, which forms the eastern boundary of the North Water Polynya, or whether they occupy other areas during parts of the Arctic winter. Further, we hypothesize that if the inner fjord system is especially attractive to narwhals, then narwhal presence will predominantly shift from the winter polynya area near our western-most recording station and into the inner fjord system as this potentially highly attractive area becomes accessible during summer.

## Methods

### Study area and acoustic data collection

**Fig. 1:**
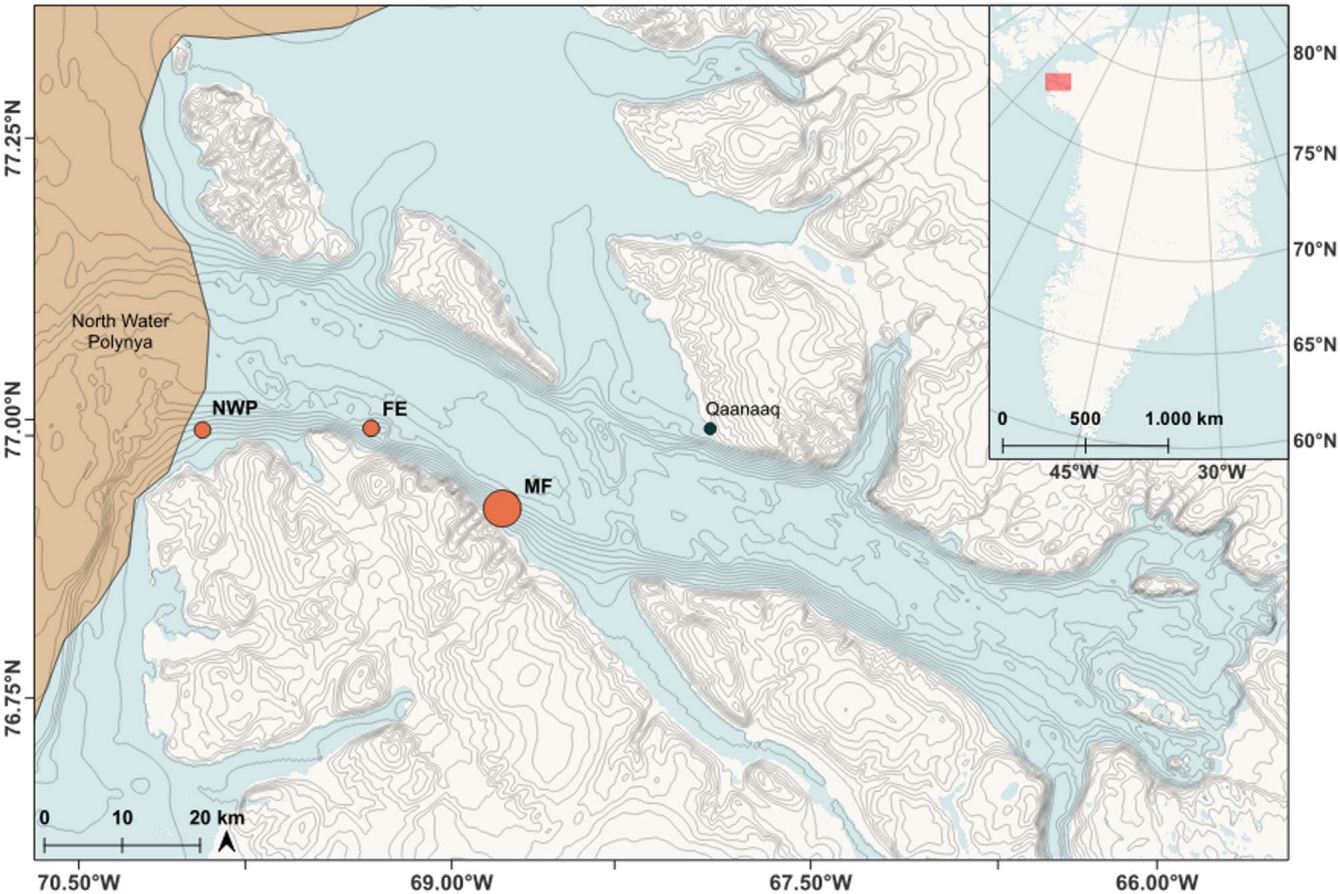
Map of Inglefield Bredning, Northwest Greenland, showing the locations of the six acoustic moorings listed in Table 1. The mid-fjord marker (MF; orange closed circle) represents four closely spaced moorings (MF01-MF04) deployed within ~ 2.5 km of one another. Contour lines indicate bathymetry at 100 m intervals derived from the GEBCO global bathymetry grid^32^. The orange-shaded area marks the North Water Polynya (Pikialasorsuaq). The inset map highlights the region’s location in Greenland. The map was created in QGIS v. 3.40.7^33^ using the CARTO Voyager No Labels basemap.

Inglefield Bredning is the primary summering habitat of the Inglefield Bredning narwhal sub-population in Northwest Greenland^7^(Fig. 1). The fjord extends roughly 100 km in length, up to 26 km in width, and exceeds 900 m in depth. It is a glacial fjord with several large marine-terminating glaciers, and it is covered by landfast sea ice from December to May/June, while it remains largely ice-free during summer and autumn.

We deployed six autonomous underwater sound recorders (SoundTrap ST600, Ocean Instruments, NZ) across Inglefield Bredning between 2022 and 2025 to capture spatial and temporal variation in narwhal acoustic activity (Table 1; Fig. 1). The mooring locations spanned from the outer fjord near the sea ice margin to the mid-fjord region. The entrance of the fjord borders the North Water Polynya (Pikialasorsuaq), where the outermost mooring (NWP01) was positioned near the polynya margin, approximately 25 km west of the fjord entrance mooring (FE01), providing year-round monitoring of conditions at the ice edge. The remaining four mid-fjord moorings (MF01-MF04) were deployed within ~ 2.5 km of each other in the central part of the fjord. This close spacing was intentional to ensure spatial consistency across years and enable direct comparison of sea-ice conditions and narwhal presence between deployment periods.

**Table 1:** Deployment details of sound recorders moored in Inglefield Bredning. All SoundTrap instruments (ST600, Ocean Instruments, NZ) recorded at a sampling rate of 192 kHz (except MF04, 128 kHz) and used duty cycles of 20–40 min/h.

Label	Full name	Start date End date	Latitude Longitude	Sample rate (kHz)	Duty cycle (min/hr)	Device depth (m) Seafloor depth (m)
FE01	Fjord-Entrance 1	2022-Jun-05 2022-Aug-02	77.2323 − 70.6568	192	40	575 590
MF01	Mid-fjord 1	2023-Sep-29 2024-Jul-15	77.2629 − 69.8541	192	20	310 320
MF02	Mid-fjord 2	2024-Apr-15 2024-Sep-23	77.2405 − 69.8448	192	20	95 106
MF03	Mid-fjord 3	2024-Sep-24 2025-Jul-11	77.2458 − 69.8427	192	20	322 330
MF04	Mid-fjord 4	2025-Apr-06 2025-Aug-07	77.3995 − 69.0730	128	20	324 330
NWP01	North-Water Polynya 1	2024-Sep-24 2025-Jul-09	77.1111 − 71.3403	192	20	315 327

Each mooring was bottom-mounted and consisted of one SoundTrap ST600 suspended in the water column at depths ranging from 95 to 575 m. The recorders had a clip level of ~ 173 dB re 1 µPa and a noise floor of 75 dB re 1 µPa RMS in the 10–80 kHz frequency band, relevant for narwhal click detection^31^. They had a flat frequency response (± 3 dB) from 20 Hz to 60 kHz and included a built-in anti-aliasing filter^31^. Additionally, each mooring was anchored with a 100 kg iron anchor connected via a 1 m rope to an acoustic releaser (PORT LF, Edgetech). Subsurface buoys (11″) provided 33–75 kg of buoyancy and were attached using 6 mm Dyneema rope. Temperature (RBRSolo^3^T, RBR Scientific) or CTD loggers (Conductivity, Temperature, and Depth, SBE37 MicroCAT, Sea-Bird Scientific) were mounted on the moorings to monitor physical environmental conditions and mooring drawdown.

The first deployment (FE01) used a 40 min/h duty cycle on the SoundTrap to increase temporal resolution during anticipated high-activity periods. In subsequent deployments, the duty cycle was reduced to 20 min/h, which still provided sufficient detection coverage while extending deployment duration by reducing power and storage demands.

To overcome the general lack of underwater acoustic data during early summer from autumn-deployments, recorders were installed in early spring just before ice breakup across four field seasons (2022–2025), as well as in September 2023 and 2024. Spring deployments were carried out in collaboration with local Inuit hunters using dog sleds on sea ice, employing a combination of power drills and traditional tools to bore through > 1 m thick ice. Autumn deployments and recoveries were also conducted in collaboration with local hunters, using small outboard boats.

### Acoustic data processing

Echolocation clicks were detected and classified using PAMGuard^34^ (v2.02.16). The “Click Detector” module identified short-duration transient signals by continuously scanning the waveform in overlapping time windows and comparing short-term energy levels against a longer-term background noise estimate. The detection threshold was set to 10 dB above the noise floor to detect as many clicks as possible without being overwhelmed by false detections. The default settings of the Click Detector module were used for all other parameters.

Detected clicks were then processed using the “Click Train Detector”, which grouped nearby clicks into click trains based on inter-click intervals (ICI settings: variance coefficient = 200 ms, min ICI = 1 ms and max ICI = 500 ms) and amplitude. Only sequences with a minimum of six clicks and a maximum train length of 2 s were retained. All additional detector settings are provided in the PAMGuard configuration file (Supplementary File S1).

To identify potential narwhal echolocation signals, a custom classifier was applied post hoc to all detected clicks. The classifier was designed to search for clicks with a peak frequency between 20 and 80 kHz, which corresponds to the typical frequency range of narwhal echolocation^35,36^. Only clicks with a duration less than 1 ms were considered. For each click, frequency bins within 6 dB of the peak amplitude were compared with adjacent control bands outside the narwhal echolocation range, and a click was classified as a narwhal signal if the peak energy exceeded the control bands by at least 6 dB.

### Sea ice data and spatial analysis

To evaluate the relationship between narwhal presence and sea-ice conditions, satellite imagery was used to determine the minimum distance between each mooring and the ice edge throughout the season. Optical imagery (mainly Sentinel-2 Level 1 C, true colour) in georeferenced 16-bit TIFF files from Sentinel Hub^37^ were imported into QGIS (version 3.40, QGIS Development Team, 2024) as raster layers, using the WGS 84/Polar Stereographic North projection. For each relevant recording date, defined as days with less than 20% cloud cover, the minimum distance from the mooring position to the ice edge was measured manually using QGIS’s built-in distance tools. Using this < 20% cloud cover threshold during recording periods, 43 images for 2022, 62 for 2023, 71 for 2024, and 129 for 2025 were obtained. Information was also saved about whether the nearest sea ice edge was in the direction of the bottom or the mouth of the fjord relative to each logger.

During the winter months (polar night, approximately October-March), when optical satellite imagery was unavailable, ice coverage was instead assessed using alternative remote sensing products, such as the cloud penetrating Sentinel-1 radar imagery based on synthetic aperture radar (SAR). These provided sub-weekly resolution comparable to the optical data. To ensure accuracy, all wintertime measurements were cross-verified against official ice charts provided by the Danish Meteorological Institute^38^. These measurements were then matched with the corresponding acoustic data to assess how narwhal presence varied with distance to the ice edge.

In addition to estimating the distance to the ice edge, we also quantified the local sea-ice concentration surrounding each mooring to capture conditions beneath and around the ice margin. Note that ice concentration (%) reflects general cover around each mooring, whereas the ice edge marks the transition to dense, continuous ice that can act as a barrier. These metrics therefore capture complementary aspects of sea-ice conditions. Daily sea-ice concentration data were obtained from the University of Bremen’s AMSR2 ASI product^39^ at 3.125 km resolution. Data for the North Water Polynya region (2022–2025) were downloaded directly from the University of Bremen archive and used to estimate the percentage of ice cover around each mooring. This complementary measure allowed us to distinguish between abrupt ice-edge retreats and more gradual thinning or partial openings in the ice cover, thereby improving the interpretation of narwhal responses to changing ice conditions.

### Estimating detection range

To quantify how far from the recorder narwhals could be acoustically detected, we first estimated the maximum horizontal acoustic detection range. This represents the distance at which a narwhal echolocation click remains detectable above background or ambient noise. We then combined this estimate with the animals’ known ability to travel horizontally beneath sea ice to evaluate whether detections recorded under ice cover could plausibly originate from narwhals.

Detection range (r) was estimated using a standard transmission loss (TL) model, assuming that clicks are detectable when the received level exceeds the noise floor by ≥ 10 dB, consistent with the PAMGuard detection settings. The effective noise floor was taken as the maximum of the environmental background noise and the SoundTrap self-noise. Inspection of the 10–80 kHz noise band showed that levels rarely fell below ~ 75 dB re 1 µPa, indicating that the system was self-noise limited. We therefore used a noise level (NL) of 75 dB re 1 µPa for the detection threshold calculation. With a narwhal click source level (SL) of 215 ± 10 dB re 1 µPa, the resulting detection threshold (DT) was 85 dB re 1 µPa (NL + 10 dB), giving: TL = SL - DT = 215 dB − 85 dB = 130 dB.

Transmission loss was modelled using:

1 $$\:TL=20\cdot{\mathrm{log}}_{10}\left(r\right)+\alpha\: \cdot r$$

where α is the absorption coefficient. Based on environmental parameters typical of the area^40^ (temperature = 1 °C, salinity = 34‰, depth = 300 m, pH = 8, frequency = 45 kHz), α was estimated to be 0.0116 dB/m. Solving Eq. 1 with TL = 130 dB yielded a maximum detection range of ~ 5 km with a likely uncertainty of ± 1 km given SL and transmission loss variations.

To account for animals swimming under the ice, we considered the maximum horizontal distance a narwhal could travel before needing to surface. Based on theoretical calculations, narwhals are estimated to travel approximately 1.2–1.4 km between breathing holes^28^. Assuming they would turn back before reaching this limit, we used 700 m as a conservative estimate of maximum under-ice travel distance.

Therefore, narwhals could theoretically be detected by the recorder even when the area is covered by landfast ice, as long as the ice edge is located no more than 5.7 km (5 km + 0.7 km) from the recorder. This estimated distance was used to evaluate the plausibility of click detections originating from narwhals beneath the landfast ice and to relate acoustic detections to the spatial position of the ice edge.

### Narwhal activity in relation to sea ice

**Fig. 2:**
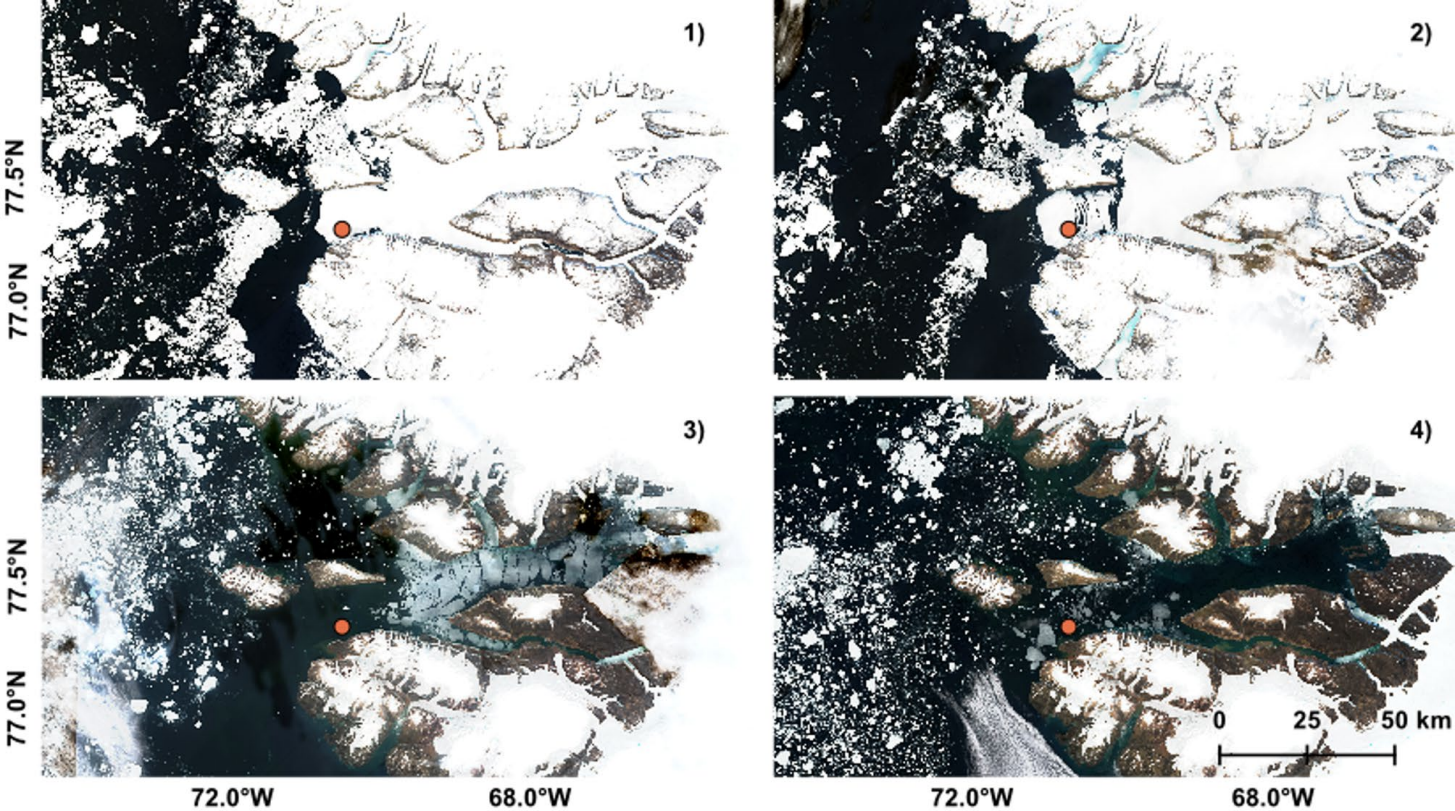
Satellite images (Sentinel-2 Level 1 C, true colour, via Sentinel Hub^37^ from 2022 illustrate the four stages of sea-ice retreat in Inglefield Bredning (orange dot = FE01). (1) Full ice cover (June 17); (2) ice edge near the mooring (June 18); (3) retreating ice (July 14); and (4) ice-free fjord (July 18). Figure layout and coordinates were created in QGIS v. 3.40.7^33^.

Narwhal acoustic presence was first determined daily (00:00–24:00 UTC) from echolocation click detections. To assess whether acoustic activity varied with changes in sea-ice conditions, sea ice in Inglefield Bredning were categorized into four stages of development based on satellite imagery: (1) full ice cover, (2) ice edge near the mooring (± 5 km), (3) retreating/reforming ice, and (4) ice-free fjord (Fig. 2). Ice retreating was defined from the first day that the consolidated ice edge began to recede into the fjord in the late spring and summer season, while ice reforming was defined from the first day in autumn or winter that new ice began to form at the fjord head and progressively advanced toward the mouth of the fjord.

Daily click detections were used as a proxy for general acoustic activity, and daily number of hours containing buzzes as a proxy for potential foraging behaviour. A buzz was defined as any second containing ≥ 50 clicks, based on manual validation showing that this threshold reliably captured sequences with the very short inter-click intervals (ICI) characteristic for foraging buzzes^41^.

### Statistical modelling

Both response variables (daily click counts and daily buzz-hours, defined as the number of hours per day containing buzz detections) were analysed using Generalized Linear Mixed Models (GLMMs) implemented in R (version 4.4.1, R Core Team, 2024) with the glmmTMB package^42,43^. A negative binomial error distribution (family = nbinom2) was chosen to account for overdispersed count data. Sea-ice stage (Period: 1–4) was included as a fixed effect, and mooring number (MooringID) was included as a random intercept to account for repeated measurements at individual moorings. For click counts, an additional observation-level random effect (1 | obs) was included to account for residual overdispersion. Model formulations were:

DetectionCounts ~ Period + (1 | MooringID) + (1 | obs).

BuzzHours ~ Period + (1 | MooringID).

Model fit and residual diagnostics were assessed using the DHARMa package^44^ (outlier test, dispersion test, and KS test on scaled residuals). DHARMa tests indicated no evidence of overdispersion (dispersion test *p* > 0.05) or excessive outliers (outlier test *p* > 0.5) for both models. Pairwise post-hoc contrasts between ice stages were conducted using estimated marginal means (EMM; emmeans package^45^ in R) with Tukey-adjusted p-values.

Because all four models (clicks/buzzes across different ice stages) showed similar residual structure and satisfactory diagnostics, only one representative diagnostic plot is provided in the Supplementary Information (Supplementary Fig. S2).

## Results

Over a three-year period (2022–2025), six bottom-moored underwater sound recorders were deployed throughout Inglefield Bredning, Northwest Greenland, providing approximately 10,020 h of actual acoustic recordings (i.e., the cumulative duration of all WAV files recorded under 20–40 min/hr duty cycles; Table 1). The number of detected clicks per day was used as a proxy for general activity, while the number of hours containing buzzes (any second containing ≥ 50 clicks) served as an indicator of potential foraging behaviour.

### Narwhal activity in relation to sea-ice concentration

**Fig. 3:**
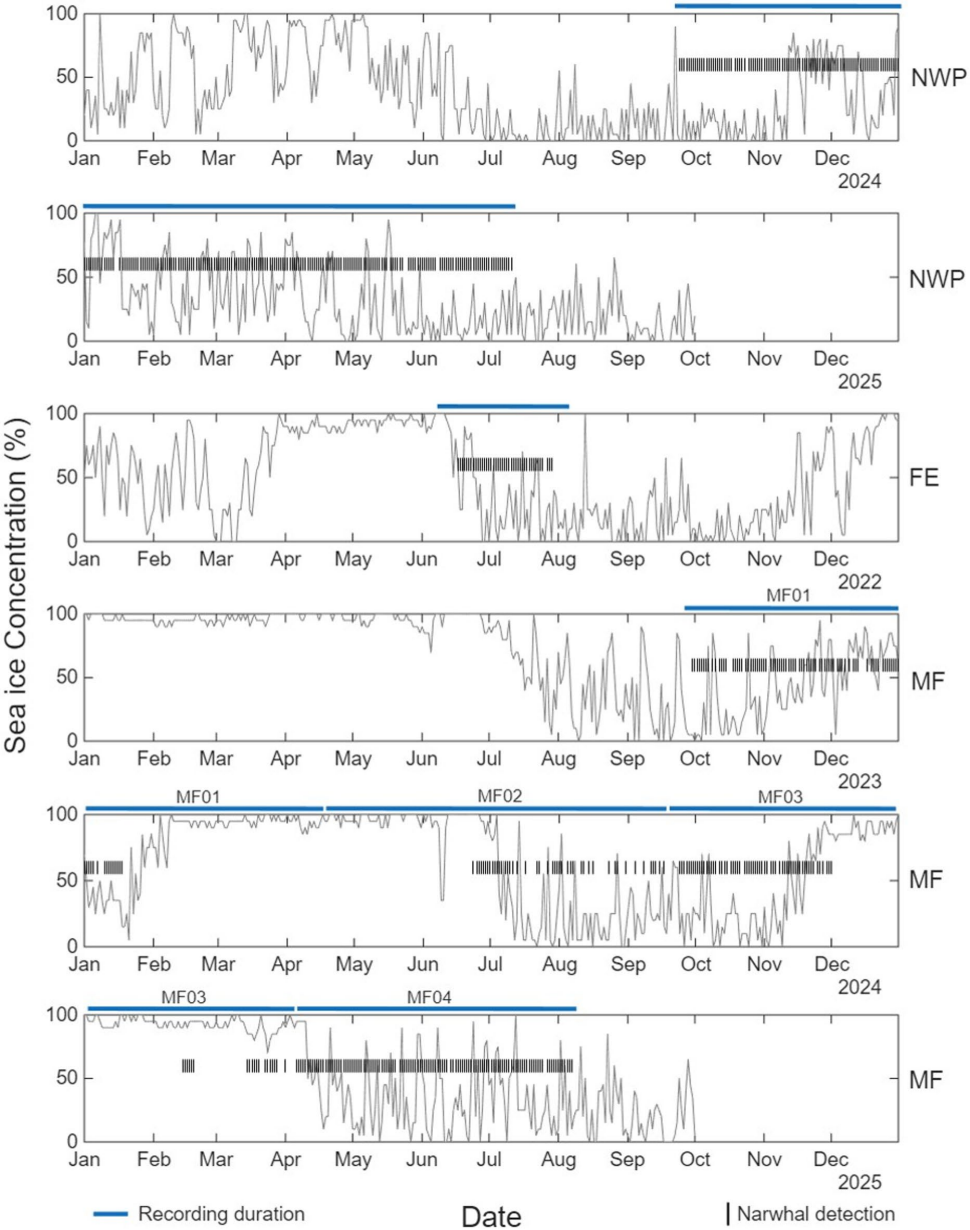
Seasonal variation in sea-ice concentration (%) and narwhal acoustic detections across all moorings: North Water Polynya (NWP01), Fjord Entrance (FE01), and Mid-Fjord (MF01-MF04). The Mid-Fjord panels combine data from four closely spaced moorings. Blue lines indicate recording periods and black, vertical bars (|) mark daily narwhal detections.

Acoustic presence and timing of narwhals between moorings (Fig. 3) reflected the recording locations within the fjord and local ice conditions.

Narwhal detections followed a seasonal pattern, primarily occurring from summer to winter. These detections coincided with periods when the ice edge was near or further into the fjord from the mooring site, indicating that large parts of the fjord were open or in the process of opening. During periods of extensive ice coverage, acoustic detections ceased entirely. Narwhal activity resumed coinciding with the seasonal retreat of the ice edge deeper into the fjord, progressively uncovering the moorings as the fjord reopened.

#### Detection range and under-ice movement

The theoretically estimated detection range of the SoundTraps for narwhal echolocation clicks was approximately 5 km. When accounting for the additional distance narwhals could swim under the ice based on dive durations and swimming speeds, the maximum detection distance extended to 5.7 km. During the study period, the maximum distance between recorders and the ice edge, at which narwhal presence was detected, was ~ 6 km in 2024 (Table 2; MF02), suggesting that detections at this distance are consistent with under-ice movement.

**Table 2:** Maximum distance (km) between stations and the sea ice edge at which narwhal acoustic presence was detected, during both the retreat and onset of sea ice.

Station	Date	Distance (km)
Retreat		
FE01_2022	2022-Jun-17	5.3
MF01_2023	2024-Jun-23	4.4
MF02_2024	2024-Jun23	6.1
MF03_2024	2024-Dec-02	5.0
MF04_2025	2024-Apr-06	3.0
Onset		
MF01_2023	2024-Jan-18	2.1
MF03_2024	2025-Mar-14	4.0
NWP01_2024	2024-Nov-19	4.0

### Narwhal response to seasonal sea ice dynamics

#### Sea-ice retreat (spring)

Acoustic activity increased markedly during the progression of sea-ice retreat. Mean click detections rose from 3,048 ± 4,133 (mean ± s.d.) counts per day under full ice coverage (stage 1; *n* = 192) to 50,434 ± 41,155 counts per day during retreating ice (stage 3; *n* = 407), before decreasing again in the ice-free stage (stage 4; 18,400 ± 13,265; *n* = 196).

**Fig. 4:**
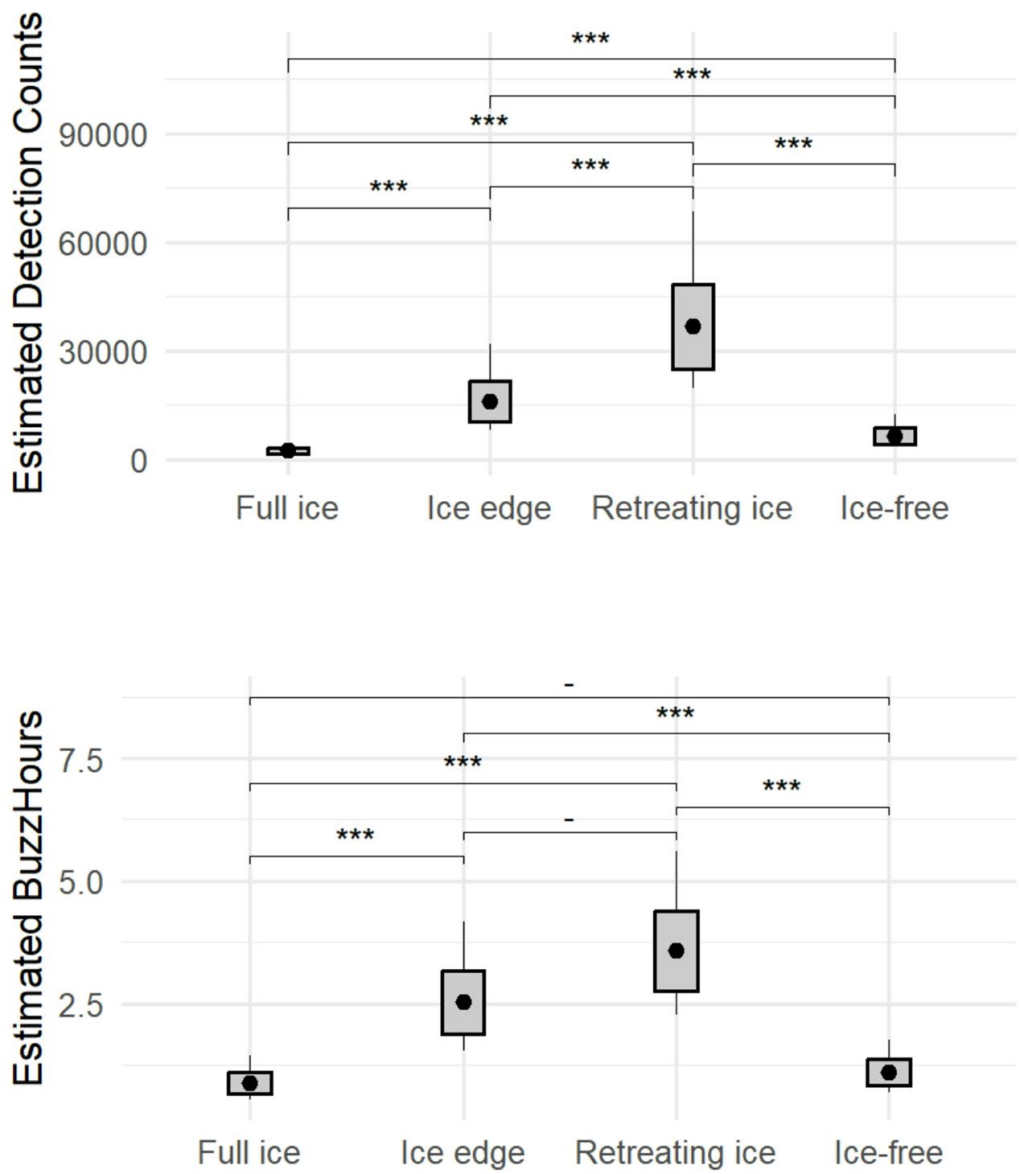
Estimated mean (± s.e.m.) daily click detections (top) and daily buzz-hours (bottom) across four stages of sea-ice retreat, derived from negative binomial GLMMs. Points (●) represent modelled means for each stage (1: full ice cover, *n* = 192, 2: ice edge near mooring (± 5 km), *n* = 91, 3: retreating ice, *n* = 407, 4: ice-free, *n* = 196). Only days belonging to the overall retreating season (when the fjord-wide ice edge was moving landward) were included in this analysis. Test symbols: *** for *p* < 0.001, ** for *p* < 0.01, * for *p* < 0.05, and a line (-) if not significant.

A negative binomial GLMM indicated a significant effect of sea-ice stage on narwhal click detections (χ^2^ = 202.48, *p* < 0.001). All tests were conducted at α = 0.05, two-tailed. EMM showed that detection rates were significantly higher during stages 2 (β = 0.91 ± 0.20, *p* = 8 × 10^− 6^) and 3 (β = 1.73 ± 0.17, *p* < 2 × 10^− 16^) compared to both full ice and ice-free conditions (all *p* < 0.001; Fig. 4). Similarly, buzz activity varied significantly with sea-ice stage (χ^2^ = 195.06, *p* < 0.001). Estimated means indicated low activity under full ice cover and highest activity during retreating ice (Fig. 4).

#### Sea-ice onset (autumn)

During the seasonal advance of sea ice, mean click detections increased from 24,052 ± 24,302 in the ice-free stage (stage 4; *n* = 147) to 38,118 ± 39,035 during reforming ice (stage 3; *n* = 132), before decreasing to 3,402 ± 14,910 under full ice coverage (stage 1; *n* = 97).

**Fig. 5:**
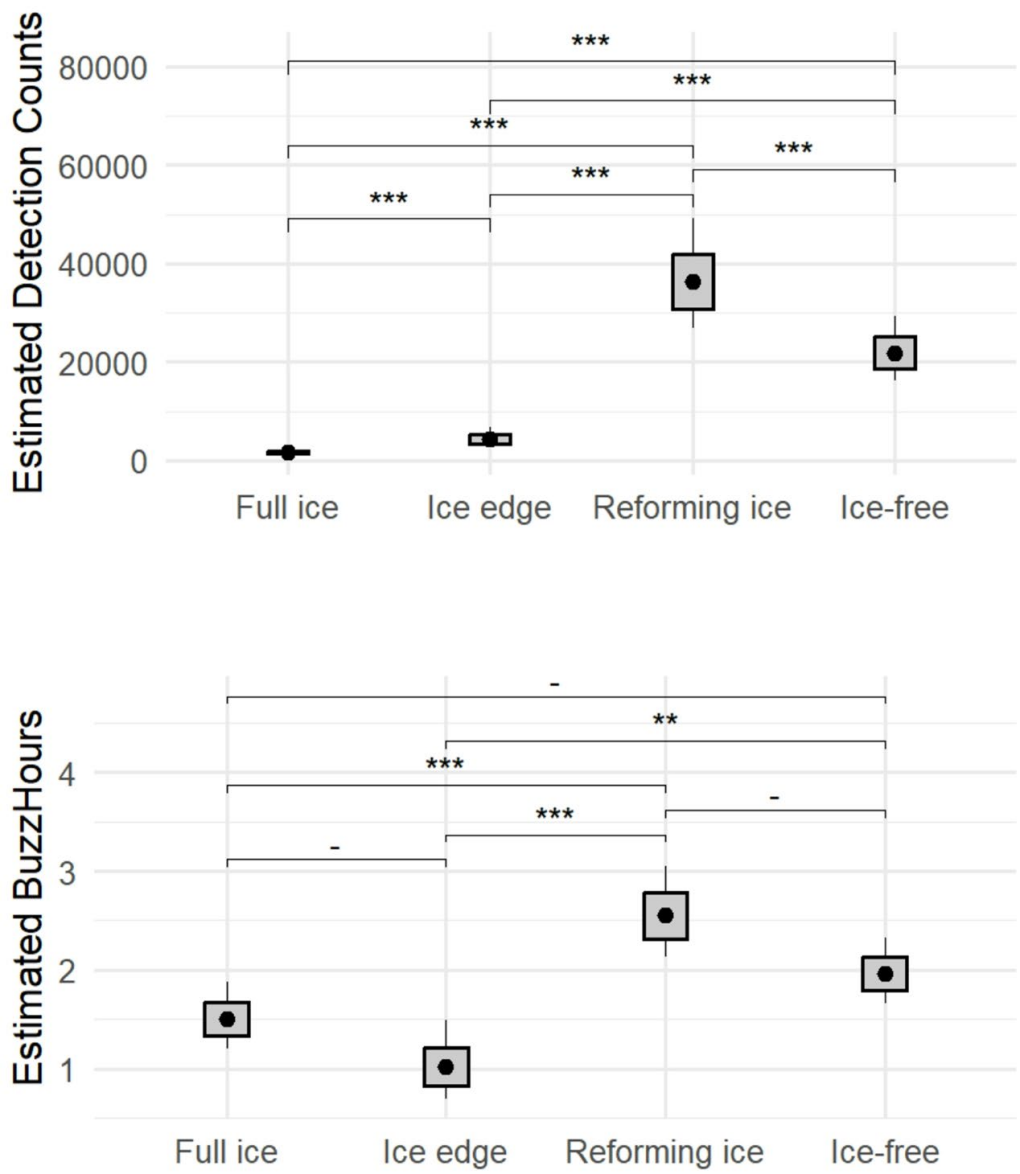
Estimated mean (± s.e.m.) daily click detections (top) and daily buzz-hours (bottom) across four stages of sea-ice onset, derived from negative binomial GLMMs. Points (●) represent modelled means for each stage (1: full ice cover, *n* = 97, 2: ice edge near mooring (± 5 km), *n* = 38, 3: reforming ice, *n* = 132, 4: ice-free, *n* = 147). Only days belonging to the overall reforming season (when the fjord-wide ice edge was advancing toward the fjord mouth) were included in this analysis. Test symbols: *** for *p* < 0.001, ** for *p* < 0.01, * for *p* < 0.05, and a line (-) if not significant.

The GLMM for click detections revealed a significant effect of sea-ice stage (χ^2^ = 254.42, *p* < 0.001). EMM showed that click rates were significantly lower during full ice (stage 1; β = -2.56 ± 0.16, *p* < 2 × 10^− 16^) and partial formation (stage 2; β = -1.63 ± 0.23, *p* < 2 × 10^− 16^) compared to reforming (stage 3; β = 0.51 ± 0.15, *p* = 0.0005) and ice-free conditions (stage 4; all *p* < 0.001; Fig. 5). Buzz activity was also significantly influenced by ice stage (χ^2^ = 228.48, *p* < 0.001). Estimated means indicated that buzzing decreased sharply from reforming stages to full ice but showed no difference between reforming and ice-free periods (*p* = 0.88; Fig. 5).

## Discussion

It is known that ice conditions affect the movement of narwhals^2,17^, however, detailed observations during ice-covered months are scarce. Extending acoustic monitoring into these months allowed us, to quantify the timing of narwhal arrivals in Inglefield Bredning relative to sea-ice retreat, providing novel insight into their seasonal fjord use. Our results show that narwhals have a year-round presence near the fjord entrance, which constitutes the eastern part of the North Water Polynya during winter (Fig. 3), with detections occurring deeper into the fjord almost immediately after sea-ice retreat and with detections ceasing a few weeks before the fjord becomes near completely ice-covered. This demonstrates that some narwhals take the first available opportunity to venture into the fjord, while others remain near the fjord entrance, thus showing that the entire fjord system is likely important to narwhals whenever the ice conditions allow them access.

This close temporal coupling between narwhal detections and ice retreat is consistent with the hypothesis that sea-ice conditions play a major role during the seasonal movements of this subpopulation^3^. Detections at all mooring sites increased sharply immediately once local openings in the ice formed, showing that narwhals appeared as soon as passage became possible (Fig. 3). At the outermost station (NWP01, located near the fjord mouth), detections were nearly continuous throughout the year, indicating that some individuals remain near the fjord mouth year-round, waiting at the ice edge and moving into the fjord as soon as conditions allow. However, a few individuals appear to stay at the fjord mouth throughout the entire year. This behaviour implies that the timing of fjord entry is driven by the immediate availability of navigable openings in the ice. Such responsiveness highlights the adaptability of narwhals to small-scale changes in sea-ice conditions and emphasizes the need to consider fine-scale habitat accessibility in future studies of Arctic cetacean movement ecology.

The several detections of narwhals at times when the fjord was near completely ice-covered indicates that narwhals routinely follow small openings into ice-packed areas, consistent with earlier behavioural observations^17,28^. In this study, detections were recorded even when the ice edge extended beyond the estimated maximum acoustic detection range of approximately 5–6 km (Table 2), which, combined with the fact that detections ceased at distances corresponding to their estimated diving capacities^28^, suggest that narwhals actively move under the ice to access these openings. Local hunters have also observed narwhals swimming beneath the consolidated ice edge during foraging^46^, further supporting their use and interaction with the marginal ice zone.

Narwhal acoustic activity varied significantly across sea-ice stages during both retreat and onset (Figs. 4 and 5). Acoustic activity increased markedly as soon as the ice edge was near the mooring, reaching peak levels when the ice retreated further into the fjord, before declining again in ice-free conditions (Fig. 4). This pattern suggests that narwhals are most active when partial ice cover remains, a period that likely offers both foraging opportunities and protection from predators such as killer whales, which are less efficient hunters in dense ice^18^. In particular, narwhal activity peaked during sea-ice stages 2 and 3, when ice was retreating but not yet absent, suggesting that individuals moved rapidly toward glacial fronts as soon as passageways opened. Thus, while ice acts as a physical barrier, its retreat also exposes previously inaccessible foraging areas, providing new feeding opportunities for narwhals as highlighted by the link between the sea-ice edge and narwhal acoustic activity.

The high click and buzz activity observed during the retreating-ice stage (Fig. 4) suggests that the marginal ice zone offers favourable foraging conditions in spring. This notion is further supported by the fact that juvenile polar cod rely on the underside of sea ice from the larval stage until roughly 10–25 cm, after which they descend to deeper waters^19–21^. This size class matches that commonly found in narwhal stomachs^46,47^, suggesting that narwhals target juvenile polar cod associated with the underside of sea ice. Although our acoustic data do not identify foraging depth, the temporal overlap between juvenile polar cod concentrations and increased narwhal buzzing points to a possible ecological link, suggesting that the retreating ice edge may offer important foraging opportunities during the transition to glacial fjord habitats.

The decline in narwhal click activity during the ice-free stage (Figs. 4 and 5) possibly reflects that many narwhals had moved further into the fjord, where foraging opportunities are favourable^11^. While our acoustic data cannot resolve individual movements, observations from local hunters and previous studies support the idea that the narwhals aggregate near the glacial fronts when the fjord is ice-free^11,46,48,49^. In contrast, during the autumn onset, activity at the moorings was markedly lower when ice formation reached the stations compared to the spring retreat (Figs. 4 and 5), suggesting that narwhals begin to leave the fjord before access becomes limited by ice, likely to avoid entrapment as ice forms. This asymmetry between retreat and onset may imply that narwhals respond not only to habitat accessibility but also to the risk of entrapment, which can cause mortality events in summering grounds^50^, perhaps leading to a strong selective pressure to leave before freeze-up. Whether this movement constitutes a true seasonal migration, or is primarily a response to ice formation constraining habitat, remains unclear. Similar observations from Fram Strait suggest that some narwhal populations may remain resident year-round in areas with favourable conditions, rather than following a strict migratory pattern^17^.

Looking ahead, the strong fidelity of narwhals to fjord environments has important ecological and conservation implications in a warming Arctic. Continued sea-ice retreat could make fjords accessible year-round, potentially reducing the seasonal displacement that currently drives narwhal movements between summering and offshore winter habitats. With climate change, many marine species are shifting their ranges poleward as oceans warm and open water days increase, tracking their preferred habitat conditions^51,52^. Although narwhals are capable of making these adjustments to their seasonal range, other studies indicate that they consistently select glacial fronts and bathymetric features over sea ice^11,12^. This is consistent with our results showing that individuals enter the fjord as soon as passageways open and only leave when forced by ice. While increased access to glacial fronts could provide stable foraging opportunities, a shift to year-round presence in the inner fjord may also have ecological and genetic consequences. Today, narwhals from multiple summering areas converge at shared wintering grounds where mating likely occurs; diminished movement between fjords and offshore habitats could therefore reduce gene flow and increase genetic isolation, a concern given the species’ already low genetic diversity^29,53^.

A shift to year-round narwhal presence in the inner fjord may alter predator-prey dynamics and habitat use, as the fjord is normally only seasonally accessible, unlike the outer polynya where narwhals already occur year-round. Such a shift could alter predation pressure on key prey such as Greenland halibut (*Reinhardtius hippoglossoides*), squid (*Gonatus fabricii*), and polar cod, which underpin Arctic food webs and support subsistence fisheries^54^. Although such effects are likely localized and self-limiting, they highlight the potential for trophic imbalances as ecosystems shift. Polar cod, in particular, depend on sea ice throughout their life cycle^19^; declining ice cover has already been linked to reduced recruitment in the Barents Sea^55^, suggesting that similar processes could reduce prey availability and affect ecosystem stability in Greenlandic waters.

Finally, while sea-ice loss is advancing rapidly, the retreat of marine-terminating glaciers may reshape fjord productivity on longer timescales. As glaciers retreat onto land, reduced freshwater and nutrient input could diminish biological productivity^10^. Whether narwhals can adapt to such transformed habitats remains uncertain, as their strong site fidelity may limit flexibility in a changing Arctic.

In conclusion, this study presents the first high-resolution acoustic documentation of narwhal arrival in their summering habitat, showing that narwhals enter the fjord almost immediately after sea ice break-up. Their presence closely follows the retreating ice edge for at least a subset of individuals, suggesting that ice access may facilitate, rather than universally trigger, spring movements within the broader summer habitat. The marginal ice zone appears to serve not just as a corridor but also as an important foraging area, likely due to seasonal prey availability such as polar cod. Narwhal acoustic activity was highest when ice was near the recorders or during retreating/reforming stages but decreased once the fjord became ice-free. This likely reflects that narwhals move further into the fjord towards glacier fronts, so lower activity near the recorder indicates a shift in position rather than reduced overall activity, highlighting glacial fjords as critical summer habitats.

Furthermore, this study demonstrates the utility of passive acoustic monitoring as a less-invasive approach for studying Arctic cetaceans in remote fjord systems. While the method cannot resolve individual movements, it provides high-resolution, year-round data under conditions where traditional satellite tagging or visual observations are challenging. By linking acoustic data with sea-ice dynamics, we show that narwhal habitat use is strongly influenced by ice access. Based on these observations, continued sea-ice decline could lead to longer residency in fjords, with potential implications for seasonal movements and gene flow. To fully resolve these patterns, future studies tracking individuals or groups will be needed to determine whether narwhals remain in distinct areas or mix more dynamically within the fjord. Investigating foraging patterns and the ecological consequences of altered residency, including shifts in predator-prey dynamics, could help determine how changes in fjord access influence narwhal foraging and interactions with prey and predators. Additionally, long-term studies are needed to assess how glacier retreat and associated changes in fjord productivity may impact narwhal habitat use. Understanding these shifts is vital for assessing the resilience of narwhal populations in a rapidly changing Arctic.

## Supplementary Information

Below is the link to the electronic supplementary material.

Supplementary Material 1

Supplementary Material 2

## Data Availability

The datasets used and/or analysed during the current study are available on reasonable request from Malene Simon (Greenland Climate Research Centre, Greenland Institute of Natural Resources; MaSi@natur.gl) and are also available from the corresponding author on reasonable request.
