# Supplementary material for: Sea ice dynamics structure narwhal presence and seasonal movements in a Northwest Greenland fjord system
Source: Sci Rep. 2026 May 21;16:23172. doi: 10.1038/s41598-026-53787-8 (PMC13396387; doi:10.1038/s41598-026-53787-8)
Supplement: Supplementary file 1 — Supplementary Material 1 [file 41598_2026_53787_MOESM1_ESM.pdf]

Supplementary information

Supplementary File S1: PAMGuard configuration file (.psfx) containing all detector settings used for click and click-train processing described in the Methods.

Supplementary Figure S2:

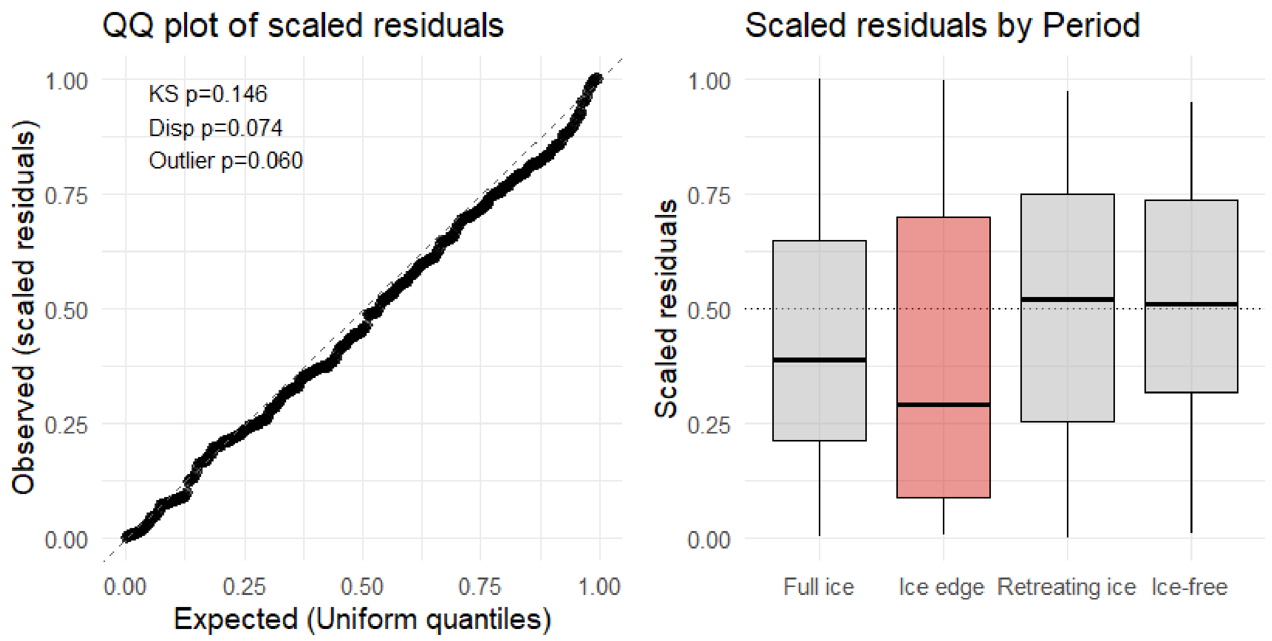

Figure S2: Model diagnostics for the Generalized Linear Mixed Model (GLMM) examining the effect of sea-ice stage on narwhal click activity. Residuals simulated with the DHARMa package show no signs of overdispersion or outlier inflation, indicating satisfactory model fit.
